# Supplementary material for: The interactome of intact mitochondria by cross-linking mass spectrometry provides evidence for coexisting respiratory supercomplexes
Source: Mol Cell Proteomics. 2017 Dec 8;17(2):216–32. doi: 10.1074/mcp.RA117.000470 (PMC5795388; doi:10.1074/mcp.RA117.000470)
Supplement: Supplemental Data [file supp_RA117.000470_133922_0_supp_23978_fzffwf.zip › spectra_annotation/mito_DR_spectra_annotation/490-1-11-1-11-1.pdf]

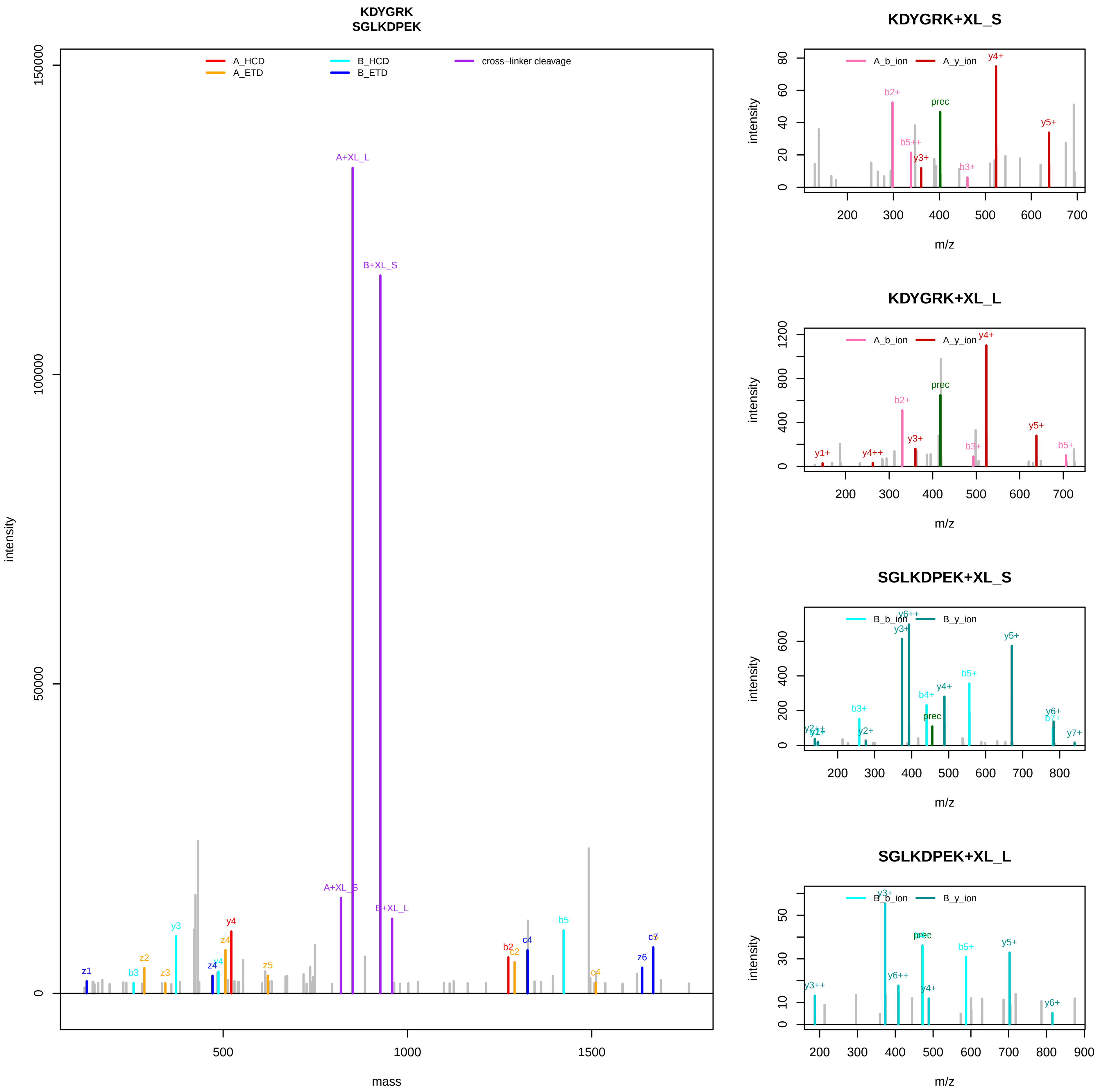
KDYGRK
SGLKDPEK
A_HCD
A_ETD
B_HCD
B_ETD
cross−linker cleavage
A+XL_L
B+XL_S
A+XL_S
B+XL_L
intensity
mass
0
50000
100000
150000
500
1000
1500
z1
b3
z2
z3
y3
z4
y4
z5
b2
c2
c4
b5
c4
z6
c7
KDYGRK+XL_S
KDYGRK+XL_L
SGLKDPEK+XL_S
SGLKDPEK+XL_L
A_b_ion
A_y_ion
B_b_ion
B_y_ion
prec
b2+
b3+
b5+
b5++
y1+
y3+
y4+
y4++
y5+
b4+
b7+
y2+
y3++
y6+
y6++
y7+
m/z
intensity
